# Picture fuzzy soft Bonferroni mean aggregation operators and their applications

**DOI:** 10.1016/j.heliyon.2023.e17278

**Published:** 2023-06-16

**Authors:** Xiaopeng Yang, Tahir Mahmood, Jabbar Ahmmad

**Affiliations:** a Department of Mathematics and Statistics, Hanshan Normal University, China; b Department of Mathematics and Statistics, International Islamic University Islamabad, Pakistan

**Keywords:** Picture fuzzy soft set, Bonferroni mean aggregation operators, Medical diagnosis

## Abstract

Due to more advanced features of the picture fuzzy soft set and valuable characteristics of aggregation operators, in this article, we present the notion of picture fuzzy soft Bonferroni mean aggregation operators and weighted picture fuzzy soft Bonferroni mean aggregation operators. Moreover, some basic properties of these introduced aggregation operators have been given. As cancer is one of the most rapidly increasing diseases globally. But due to different kinds of cancer diseases, it is very difficult to say which type of cancer disease is increasing rapidly. So to reduce this difficulty in the medical field, we have applied our work to medical diagnosis problems to ensure that fuzzy ideas can also help in the medical field. Also, an algorithm along with a descriptive example is established that conforms to the authenticity of initiated work. Furthermore, a comparative analysis of the introduced work has been given to show how this work is more efficient and dominant than other existing theories.

## 1 Introduction

In the past few decades, medical diagnosis has become a more complicated field due to the complexities of different diseases of the same type. For example, if we discuss the different types of cancer disease, then it is very difficult to say which type of cancer disease is increasing rapidly in which is not. In this situation, the fuzzy set (FS) theory introduced by Zadeh [1] opens the door for researchers to find the applications of their work in the medical field. So, many researchers applied FS theory to the medical field like Ahn et al. [2] proposed a fuzzy differential diagnosis of headaches by applying different methods. Moreover Innocent and John [3] established computer-aided fuzzy medical diagnosis and show how FS theory works in this field. Also, De et al. [4] provide the application of an intuitionistic fuzzy set (IFS) to a medical diagnosis that is a more generalized structure. Furthermore, Khatibi and Motazer [5] proposed a comparison of IFS and FS in medical diagnosis. Mahmood et al. [6] also find the applications of their work in medical diagnosis problems. We can see from the above analysis that fuzzy set theory has an effective and valuable range of applications in the medical field.

As aggregations operators (AOs) are the fundamental apparatus to tackle the FS theory because, with the help of AOs, a large number of data can be converted to a single number that can further help in the selection of different decision-making problems. So, Yager [7] introduced some generalized Bonferroni mean aggregation operators. The fuzzy set theory opens a new path for researchers and diverts their attention to this field. Due to the huge range of applications of FS theory in mathematical programming [8], power systems [9], and production management [10], researchers started working on fuzzy set theory. Although FS is a valuable structure it can consider only one aspect in its structure like membership grade (MG), but in many decision-making problems, researchers observe that in many circumstances we have to discuss two-dimensional grades like membership grades and non-membership grades in one structure. So, Yager solve this problem and introduced the term IFS [11] which can consider the MG and non-membership grade (NMG) in one structure. So, IFS is a more generalized structure than that FS. Due to the more advanced structure, researchers produce some mathematical structures based on IFS like Chaira and Ray [12] produce some new measures using the setting of IFS and find their uses to edge identification. Moreover, Xu [13] defines some AOs for IFS. Also, Xu and Yager [14] produced some Bonferroni mean aggregation operators (BMAOs) for IFS. Furthermore, Wang and Liu [15] established the geometric AOs under the environment of Einstein operations. Note that IFS uses the necessary condition that the sum (MG, NMG) must belong to [0, 1] but in many situations, IFS fails when decision-makers (DMs) take 0.5 as MG and 0.6 as NMG then 0.5+0.6∉[0,1]. It means that IFS is a limited structure. So, there is a need to generalize this notion to some other notion that can handle that situation. So, Yager [16] again produces the structure of the Pythagorean fuzzy set (PyFS). In this structure, we can note that Yager uses the condition that $\left({MG}^{2},{NMG}^{2}\right)\in \left[0,1\right]\text{.}$ It means that $0.5^{2}+0.6^{2}\in \left[0,1\right]$ that shows that data that cannot be handled by IFS can be solved by PyF information. Many new theories have been developed based on PyFS like Garg [17] introduced the new logarithmic laws for PyFS and produce their AOs for its application. Moreover, Zheng et al. [18] introduced some generalized PyF Bonferroni means AOs and finds its application to DM issues. Also, PyF Bonferroni means AOs, and the algorithm with multithreading is introduced by Ling et al. [19]. Some researchers introduce some new mathematical structures for PyFS like similarities measure and distance measure has been introduced by Peng [20]. Also, Peng et al. [21] introduced the PyF information measure. Ullah et al. [22] introduced some new distance measures for complex PyFS and utilize this notion in pattern recognition. Also, Akram et al. [23] initiated the group DM using the setting of the PyF TOPSIS method. Note that PyFS is also a limited structure because in many situations its necessary condition fails to hold like when we have to face 0.8 as MG and 0.9 as NMG then note that $0.8^{2}+0.9^{2}\notin \left[0,1\right]\text{.}$ it means researchers observe that there is a need to introduce a stronger notion that can handle this situation. So, Yager [24] initiated the setting of q-rung orthopair fuzzy set (q-ROFS) that use uses further strong constraint that $sum\;\left({MD}^{q}+{NMD}^{q}\right)\in \left[0,1\right]\text{.}$ So, many researchers utilize this notion in many filed like social network group DM [25] and sustainable energy planning decision management [26]. Many new methods have been developed like the MABAC method has been initiated by Wang et al. [27]. Moreover, Liu and Liu [28] introduced the BMAOs and provide their applications to DM. All these notions have some inherent limitations.

Molodtsov [29] initiated a novel structure of a soft set $\left(S_{ft}S\right)$ as an advanced mathematical structure for handling ambiguity. Note that $S_{ft}S$ can generalize all the above-given theories because it can consider the parameterization toll. Ali et al. [30] established some new laws under the setting of $S_{ft}S$. Also, Ali et al. [31] initiated some new notions of $S_{ft}S$ linked with new operations. Many researchers try to find out the combined structure of $S_{ft}S$ along with other FS notions and some new notions are successfully obtained like a fuzzy soft set $\left(FS_{ft}S\right)$ [32], IF soft set $\left(IFS_{ft}S\right)$ [33], PyF soft set $\left(PyFS_{ft}S\right)$ [34] and q-ROF soft set $\left(q-ROFS_{ft}S\right)$ [35]. Furthermore, by using these theories many AOs, similarity measures, and some new methods have been developed to cover ambiguous and uncertain data in many DM issues. Arora and Garg [36] established AOs for $IFS_{ft}S$. Moreover, some BMAOs based on $IFS_{ft}$ settings have been discovered by Garg and Arora [37]. $PyFS_{ft}$ multi-criteria group decision-making (MCGDM) methods under TOPSIS, VIKOR, and AOs have been introduced by Naeem et al. [38].

Observe that when decision-makers have to tackle three types of aspects like yes, no, and abstain then all above-given theories fail to tackle such kind of information. So, there is a need to form such a type of structure for which all these three types of possibilities have been covered. So Cuong [39] produced the notion of a picture fuzzy set (PFS) that can consider the MG, NMG, and abstain grade (AG) with the condition that sum(MG,AG,NMG)∈[0,1]. PFS is a more generalized structure and based on this notion many ideas have been developed. Wei [40] initiated PF aggregation operators and their uses in MCDM. Moreover, Akram et al. [41] combine PFS with complex FS theory and developed DM under complex PF Hamacher AOs. Some PFBM aggregation operators have been developed by Ates and Akey [42]. Ashraf et al. [43] proposed different approaches to MCGDM problems for the PF environment.

The hybrid notion of picture fuzzy soft set $\left(PFS_{ft}S\right)$ have been introduced by Yang et al. [44] that can generalize all the above-given notions. The idea of the picture fuzzy soft set is more advanced because it can cover the MG, NMG and AG in one structure. Also, the picture fuzzy soft set can consider the parameterization tool and this property ranked this structure more unique from other prevailing theories of intuitionistic fuzzy set and picture fuzzy set. Picture fuzzy soft is the dominant structure and it can handle many real-life issues. For instant when we analyze the phenomenon of voting, we can vote someone, vote against someone, abstain from voting, or refuse to vote. Similarly, modeling ambiguity has recently grown among scholars. $PFS_{ft}$ structures are generally employed when several human responses exist like yes, no, abstain, and refusal. For example, a visit from an administrative worker of a company could be an appropriate example of $PFS_{ft}S\text{.}$ Some staff members wish to visit two different countries: Italy and England. However, certain workers prefer to travel to Italy (MG) instead of England (NMG), while others desire to trip to both Italy and England, i.e., neutral personnel. However, some employees, referred to as refusal grades, do not wish to attend both locations. We can observe that $PFS_{ft}S$ is the structure that can handle such kind of situations and it can consider all of these three kinds of aspects like MG, AG, and NMG in one structure with the condition that sum(MG,AG,NMG)∈[0,1]. It means that the prevailing theories of the intuitionistic fuzzy soft set, Pythagorean fuzzy soft set, and q-rung orthopair fuzzy soft have some drawbacks that they cannot consider the AG in their structure. Also existing notions of $PFS_{ft}S$ have the advantage over simple picture fuzzy set because $PFS_{ft}S$ can cover the parameterization tool. When we use only one parameter then $PFS_{ft}S$ degenerate onto a simple picture fuzzy set. So from the above observation, we can say that the existing notion is dominant to prevailing theories.

The point-wise contribution of this study is given by

1. First of all, based on the advanced notion of $PFS_{ft}S$, we have developed the fundamental laws of sum, product, scalar multiplication, and scalar power for picture fuzzy soft numbers.
2. As aggregation operators are the basic tools that can convert complex data into a single value. So aggregation operators can help in many decision-making problems. So based on the explored operational laws for $PFS_{ft}Ns\text{,}$ we have developed the Bonferroni mean aggregation operators like picture fuzzy soft Bonferroni mean aggregation operators.
3. Moreover, we have developed the notion of weighted picture fuzzy soft Bonferroni mean and weighted picture fuzzy soft Bonferroni mean aggregation operators.
4. Basic properties like idempotency, boundedness, monotonicity, and commutativity have been proved for these developed aggregation operators.
5. Moreover, to show the utilization of these developed notions, we have provided an algorithm and used these notions in medical diagnosis that show that the developed notions play a vital role in the medical field.
6. In the end, we have explored the comparative analysis of the developed notion by comparing the developed notion with some prevailing theories to show the advantages and superiority of the established work.

Our article is formulated in this fashion, in section 1 we have discussed the definitions of $S_{ft},S{FS}_{ft}S$, PFS, ${PFS}_{ft}S$ and their fundamental laws. Section 3, elaborate $PFS_{ft}BM$ aggregation operator, and their properties. In section 4, we have initiated $WPFS_{ft}BM$ aggregation operator. Section 5 discusses the different cases of the introduced work. Section 6 deals with decision-making algorithms that show the authenticity and reliability of initiated work. In section 7, we have discussed the comparative analysis of initiated work to show how this initiated work is more efficient than other existing theories. At the end of section 8, we have given conclusion remarks.

## 2 Preliminaries

Fuzzy set theory can formalize the uncertain data on which medical diagnosis and treatment are based. Firstly, it elaborates on the medical entries in the form of fuzzy sets, and secondly, it provides a linguistic approach. Finally, the fuzzy set theory provides reasonable methods that can handle these issues in medical diagnosis as given in Ref. [3]. All these facts provide the information that fuzzy set theory might be one of the fundamental tools for the development of computerized medical diagnosis systems.

In the following, we studied some definitions of $S_{ft}S$, ${FS}_{ft}S$, PFS, ${PFS}_{ft}S$ and their operational laws.

Definition 1 [29] Let $\bar{ͳ}$ denote parameter set. A pair $\left(\bar{ͳ},\bar{Ҋ}\right)$ is a soft set over $\hat{ṹ}$ where $\bar{ͳ}$ is a function from $\bar{Ҋ}$ to $P^{\hat{ṹ}}$ and $P^{\hat{ṹ}}$ denote the power set of $\hat{ṹ}$.

Definition 2 [32] A pair $\left(\bar{ͳ},\bar{Ҋ}\right)$ is called fuzzy soft set over $\hat{ṹ}$ where $\bar{ͳ}:\bar{Ҋ}⟶{\bar{ͳ}}^{\hat{ṹ}}$, and ${\bar{ͳ}}^{\hat{ṹ}}$ denote the power set of FS of $\hat{ṹ}$. Now for $e_{ԟ}\in \bar{Ҋ}$, $FS_{ft}S$ is given by

$${\bar{ͳ}}_{e_{ԟ}}\left({ѷ}_{ϛ}\right)=\left\{\left({ѷ}_{ϛ},G_{ԟ}\left({ѷ}_{ϛ}\right)|\;{ѷ}_{ϛ}\in \hat{ṹ}\right)\right\}$$

Where $G_{ԟ}\left(ѷ\right)$ present the membership grade (MG).

Definition 3 [44] A pair $\left(\bar{ͳ},\bar{Ҋ}\right)$ is known as a picture fuzzy soft set if $\bar{ͳ}:\bar{Ҋ}⟶P{\bar{ͳ}}^{\hat{ṹ}}$, where $P{\bar{ͳ}}^{\hat{ṹ}}$ present the power set of picture fuzzy subsets of $\hat{ṹ}$. Now for $e_{ԟ}\in \bar{Ҋ}$, the $PFS_{ft}S$ is given below

$${\bar{ͳ}}_{e_{ԟ}}\left({ѷ}_{ϛ}\right)=\left\{\left({ѷ}_{ϛ},G_{ԟ}\left({ѷ}_{ϛ}\right),O_{ԟ}\left({ѷ}_{ϛ}\right),{ȿ}_{ԟ}\left({ѷ}_{ϛ}\right)|\;{ѷ}_{ϛ}\in \hat{ṹ}\right)\right\}$$

Where $G_{ԟ}\left({ѷ}_{ϛ}\right)$, $O_{ԟ}\left({ѷ}_{ϛ}\right)$ and ${ȿ}_{ԟ}\left({ѷ}_{ϛ}\right)$ present MG, AG, and NMG respectively with $sum\left(G_{ԟ}\left({ѷ}_{ϛ}\right),O_{ԟ}\left({ѷ}_{ϛ}\right){,ȿ}_{ԟ}\left({ѷ}_{ϛ}\right)\right)\in \left[0,1\right]\text{.}$ For convenience, the triplet ${\hat{ơ}}_{ϛԟ}=\left\langle G_{ϛԟ},O_{ϛԟ},{ȿ}_{ϛԟ}\right\rangle$ is called picture fuzzy soft number $\left(PFS_{ft}Ns\right)$.

Definition 4 [7] Let $Q=\left\{{\hat{ơ}}_{1},{\hat{ơ}}_{2},.\;.\;.{\hat{ơ}}_{ͷ}\right\}$ be a family of alternatives. For any real number r,s>0, the BM is given as

(1) $$B^{r,s}\left({\hat{ơ}}_{1},{\hat{ơ}}_{2},.\;.\;.{\hat{ơ}}_{ͷ}\right)={\left(\frac{1}{ͷ\left(ͷ-1\right)}\sum_{\begin{array}{c} ϛ,Ѯ=1\\ ϛ\neq Ѯ \end{array}}^{ͷ}{\hat{ơ}}_{ϛ}^{r}{\hat{ơ}}_{Ѯ}^{s}\right)}^{r+s}$$

## 3 Bonferroni mean aggregation operators based on $PFS_{ft}S$

In this portion, we introduce $PFS_{ft}BM$ operator and $WPFS_{ft}BM$ operator respectively. Also, we define some operational rules for $PFS_{ft}Ns$.

### 3.1 Operational rules for $PFS_{ft}Ns$

Definition 5 Let $\hat{\hat{pf}}=\left\langle G,O,ȿ\right\rangle$, ${\hat{\hat{pf}}}_{11}=\left\langle G_{11},O_{11},{ȿ}_{11}\right\rangle$ and ${\hat{pf}}_{12}=\left\langle G_{12},O_{12},{ȿ}_{12}\right\rangle$ be three $PFS_{ft}Ns$ and λ>0. Then we have

(i) ${\hat{pf}}_{11}⊕{\hat{\hat{pf}}}_{12}=\left\langle 1-\left(1-G_{11}\right)\left(1-G_{12}\right),O_{11}O_{12},{ȿ}_{11}{ȿ}_{12}\right\rangle$.
(ii) ${\hat{pf}}_{11}⊗{\hat{\hat{pf}}}_{12}=\left\langle G_{11}G_{12},O_{11}O_{12},1-\left(1-{ȿ}_{11}\right)\left(1-{ȿ}_{12}\right)\right\rangle$.
(iii) $\lambda \hat{\hat{pf}}=\left\langle 1-{\left(1-G\right)}^{\lambda },O^{\lambda },{ȿ}^{\lambda }\right\rangle$.
(iv) ${\hat{\hat{pf}}}^{\lambda }=\left\langle G^{\lambda },1-{\left(1-O\right)}^{\lambda },1-{\left(1-ȿ\right)}^{\lambda }\right\rangle$.

Definition 6 For $S_{ft}Ns$ ${\hat{pf}}_{ϛԟ}=\left\langle G_{ϛԟ},O_{ϛԟ},{ȿ}_{ϛԟ}\right\rangle$, the notion of score function and accuracy function is given by

(2) $$Sco.\left({\hat{pf}}_{ϛԟ}\right)=G_{ϛԟ}-O_{ϛԟ}-{ȿ}_{ϛԟ}$$

And

(3) $$Acu.\left({\hat{pf}}_{ϛԟ}\right)=G_{ϛԟ}+O_{ϛԟ}+{ȿ}_{ϛԟ}$$

Where $Sco.\left({\hat{pf}}_{ϛԟ}\right)\in \left[-1,1\right]$ and $Acu.\left({\hat{pf}}_{ϛԟ}\right)\in \left[0,1\right]$.

Note that for two $PFS_{ft}Ns$ ${\hat{ơ}}_{ϛԟ}$ and ${\hat{\beta }}_{ϛԟ}$, we have

1. if $Sco.\left({\hat{ơ}}_{ϛԟ}\right)>S\left({\hat{\beta }}_{ϛԟ}\right)$ then ${\hat{ơ}}_{ϛԟ}>{\hat{\beta }}_{ϛԟ}$.
2. if $Sco.\left({\hat{ơ}}_{ϛԟ}\right)<S\left({\hat{\beta }}_{ϛԟ}\right)$ then ${\hat{ơ}}_{ϛԟ}<{\hat{\beta }}_{ϛԟ}$.
3. if $Sco.\left({\hat{ơ}}_{ϛԟ}\right)=S\left({\hat{\beta }}_{ϛԟ}\right)$ then
(i) if $Acu.\left({\hat{ơ}}_{ϛԟ}\right)>A\left({\hat{\beta }}_{ϛԟ}\right)$ then ${\hat{ơ}}_{ϛԟ}>{\hat{\beta }}_{ϛԟ}$.
(ii) if $Acu.\left({\hat{ơ}}_{ϛԟ}\right)<A\left({\hat{\beta }}_{ϛԟ}\right)$ then ${\hat{ơ}}_{ϛԟ}<{\hat{\beta }}_{ϛԟ}$.
(iii) if $Acu.\left({\hat{ơ}}_{ϛԟ}\right)=A\left({\hat{\beta }}_{ϛԟ}\right)$ then ${\hat{ơ}}_{ϛԟ}={\hat{\beta }}_{ϛԟ}\text{.}$.

Now we define BM aggregation operators under the settings of $PFS_{ft}Ns$.

### 3.2 Picture fuzzy soft Bonferroni mean operator

In this section, we will define the structure of $PFS_{ft}BM$ aggregation operator and $WPFS_{ft}BM$ aggregation operators.

Now based on equation (1), we can extend BM operators for $PFS_{ft}Ns$ as follows

Definition 7 For a family of $PFS_{ft}Ns\;{\hat{pf}}_{ϛԟ}\;\left(ϛ=1,2,..\;.\;.,ͷ;ԟ=1,2,.\;.\;.\;.m\right)$, a $PFS_{ft}BM$ operator is a function $PFS_{ft}BM:z^{ͷ}⟶z$ given by

(4) $$PFS_{ft}BM^{r,s}\left({\hat{pf}}_{11},{\hat{pf}}_{12},.\;.\;.,{\hat{pf}}_{ͷm}\right)={\left(\frac{1}{mͷ\left(m-1\right)\left(ͷ-1\right)}\begin{array}{c} \begin{array}{c} m\\ \;⊕ \end{array}\\ \begin{array}{c} ԟ,ᶅ=1\\ ԟ\neq ᶅ \end{array} \end{array}\begin{array}{c} \begin{array}{c} ͷ\\ \;⊕ \end{array}\\ \begin{array}{c} ϛ,Ѯ=1\\ ϛ\neq Ѯ \end{array} \end{array}\left({\hat{pf}}_{ϛԟ}^{r}⨂{\hat{pf}}_{Ѯᶅ}^{s}\right)\right)}^{\frac{1}{r+s}}$$

Where r,s≥0 are real values.

Now using equation (4), we can define $PFS_{ft}BM$ operator as follows

Theorem 1 *For the family of*$PFS_{ft}Ns\;{\hat{pf}}_{ϛԟ}=\left\langle G_{ϛԟ},O_{ϛԟ},{ȿ}_{ϛԟ}\right\rangle \text{,}$*the aggregated results by applying*$PFS_{ft}BM$*operator is again a*$PFS_{ft}N$*as given by*:

(5) $$PFS_{ft}BM^{r,s}\left({\hat{pf}}_{11},{\hat{pf}}_{12},.\;.\;.,{\hat{pf}}_{ͷm}\right)=\left(\begin{array}{c} {\left(1-\left(\prod_{\begin{array}{c} ԟ,ᶅ=1\\ ԟ\neq ᶅ \end{array}}^{m}\prod_{\begin{array}{c} ϛ,Ѯ=1\\ ϛ\neq Ѯ \end{array}}^{ͷ}{\left(1-G_{ϛԟ}^{r}G_{Ѯᶅ}^{s}\right)}^{\frac{1}{mͷ\left(m-1\right)\left(ͷ-1\right)}}\right)\right)}^{\frac{1}{r+s}},\\ 1-{\left(1-\prod_{\begin{array}{c} ԟ,ᶅ=1\\ ԟ\neq ᶅ \end{array}}^{m}\prod_{\begin{array}{c} ϛ,Ѯ=1\\ ϛ\neq Ѯ \end{array}}^{ͷ}{\left(1-{\left(1-O_{ϛԟ}\right)}^{r}{\left(1-O_{Ѯᶅ}\right)}^{s}\right)}^{\frac{1}{mͷ\left(m-1\right)\left(ͷ-1\right)}}\right)}^{\frac{1}{r+s}},\\ 1-{\left(1-\prod_{\begin{array}{c} ԟ,ᶅ=1\\ ԟ\neq ᶅ \end{array}}^{m}\prod_{\begin{array}{c} ϛ,Ѯ=1\\ ϛ\neq Ѯ \end{array}}^{ͷ}{\left(1-{\left(1-{ȿ}_{ϛԟ}\right)}^{r}{\left(1-{ȿ}_{Ѯᶅ}\right)}^{s}\right)}^{\frac{1}{mͷ\left(m-1\right)\left(ͷ-1\right)}}\right)}^{\frac{1}{r+s}} \end{array}\right)$$

*Proof*: *We use the mathematical induction method to prove the above-given result*. *As for all*$ϛ,ԟ;{\hat{pf}}_{ϛԟ}$*is a*$PFS_{ft}N$*so we have*$0\leq G_{ϛԟ},O_{ϛԟ},{ȿ}_{ϛԟ}\leq 1$*and*${0\leq G}_{ϛԟ}+O_{ϛԟ}+{ȿ}_{ϛԟ}\leq 1$. *Now we follow the following steps*

Step-1 ***For***ͷ=2, *we have*

$$PFS_{ft}BM^{r,s}\left({\hat{pf}}_{11},{\hat{pf}}_{12},.\;.\;.,{\hat{pf}}_{2m}\right)={\left(\frac{1}{2m\left(m-1\right)}\begin{array}{c} \begin{array}{c} m\\ \;⊕ \end{array}\\ \begin{array}{c} ԟ,ᶅ=1\\ ԟ\neq ᶅ \end{array} \end{array}\begin{array}{c} \begin{array}{c} 2\\ \;⊕ \end{array}\\ \begin{array}{c} ϛ,Ѯ=1\\ ϛ\neq Ѯ \end{array} \end{array}\left({\hat{pf}}_{ϛԟ}^{r}⨂{\hat{pf}}_{Ѯᶅ}^{s}\right)\right)}^{\frac{1}{r+s}}$$

*Now by using definition* (6), *we get*

$$\begin{array}{c} \begin{array}{c} 2\;⊕ \end{array}\\ \begin{array}{c} ϛ,Ѯ=1\\ ϛ\neq Ѯ \end{array} \end{array}\left({\hat{pf}}_{ϛԟ}^{r}⨂{\hat{pf}}_{Ѯᶅ}^{s}\right)=\left({\hat{pf}}_{1ԟ}^{r}⨂{\hat{pf}}_{2ᶅ}^{s}\right)⊕\left({\hat{pf}}_{2ԟ}^{r}⨂{\hat{pf}}_{1ᶅ}^{s}\right)$$

$$=\left(\begin{array}{c} G_{1ԟ}^{r}G_{2ᶅ}^{s},1-{\left(1-O_{1ԟ}\right)}^{r}{\left(1-O_{2ᶅ}\right)}^{s},\\ 1-{\left(1-{ȿ}_{1ԟ}\right)}^{r}{\left(1-{ȿ}_{2ᶅ}\right)}^{s} \end{array}\right)⊕\left(\begin{array}{c} G_{2ԟ}^{r}G_{1ᶅ}^{s},1-{\left(1-O_{2ԟ}\right)}^{r}{\left(1-O_{1ᶅ}\right)}^{s},\\ 1-{\left(1-{ȿ}_{2ԟ}\right)}^{r}{\left(1-{ȿ}_{1ᶅ}\right)}^{s} \end{array}\right)$$

$$=\left(\begin{array}{c} 1-\left(1-G_{1ԟ}^{r}G_{2ᶅ}^{s}\right)\left(1-G_{2ԟ}^{r}G_{1ᶅ}^{s}\right),\\ \left(1-{\left(1-O_{1ԟ}\right)}^{r}{\left(1-O_{2ᶅ}\right)}^{s}\right)\left(1-{\left(1-O_{2ԟ}\right)}^{r}{\left(1-O_{1ᶅ}\right)}^{s}\right),\\ \left(1-{\left(1-{ȿ}_{1ԟ}\right)}^{r}{\left(1-{ȿ}_{2ᶅ}\right)}^{s}\right)\left(1-{\left(1-{ȿ}_{2ԟ}\right)}^{r}{\left(1-{ȿ}_{1ᶅ}\right)}^{s}\right) \end{array}\right)$$

$$=\left(1-\prod_{\begin{array}{c} ϛ,Ѯ=1\\ ϛ\neq Ѯ \end{array}}^{2}\left(1-G_{ϛԟ}^{r}G_{Ѯᶅ}^{s}\right),\prod_{\begin{array}{c} ϛ,Ѯ=1\\ ϛ\neq Ѯ \end{array}}^{2}\left(1-{\left(1-O_{ϛԟ}\right)}^{r}{\left(1-O_{Ѯᶅ}\right)}^{s}\right),\prod_{\begin{array}{c} ϛ,Ѯ=1\\ ϛ\neq Ѯ \end{array}}^{2}\left(1-{\left(1-{ȿ}_{ϛԟ}\right)}^{r}{\left(1-{ȿ}_{Ѯᶅ}\right)}^{s}\right)\right)$$

*Hence*

$${\left(\frac{1}{2m\left(m-1\right)}\begin{array}{c} \begin{array}{c} m\\ \;⊕ \end{array}\\ \begin{array}{c} ԟ,ᶅ=1\\ ԟ\neq ᶅ \end{array} \end{array}\begin{array}{c} \begin{array}{c} 2\\ \;⊕ \end{array}\\ \begin{array}{c} ϛ,Ѯ=1\\ ϛ\neq Ѯ \end{array} \end{array}\left({\hat{pf}}_{ϛԟ}^{r}⨂{\hat{pf}}_{Ѯᶅ}^{s}\right)\right)}^{\frac{1}{r+s}}$$

$$=\left(\begin{array}{c} {\left(1-\left(\prod_{\begin{array}{c} ԟ,ᶅ=1\\ ԟ\neq ᶅ \end{array}}^{m}\prod_{\begin{array}{c} ϛ,Ѯ=1\\ ϛ\neq Ѯ \end{array}}^{2}{\left(1-G_{ϛԟ}^{r}G_{Ѯᶅ}^{s}\right)}^{\frac{1}{2m\left(m-1\right)}}\right)\right)}^{\frac{1}{r+s}},1-{\left(1-\prod_{\begin{array}{c} ԟ,ᶅ=1\\ ԟ\neq ᶅ \end{array}}^{m}\prod_{\begin{array}{c} ϛ,Ѯ=1\\ ϛ\neq Ѯ \end{array}}^{2}{\left(1-{\left(1-O_{ϛԟ}\right)}^{r}{\left(1-O_{Ѯᶅ}\right)}^{s}\right)}^{\frac{1}{2m\left(m-1\right)}}\right)}^{\frac{1}{r+s}},\\ 1-{\left(1-\prod_{\begin{array}{c} ԟ,ᶅ=1\\ ԟ\neq ᶅ \end{array}}^{m}\prod_{\begin{array}{c} ϛ,Ѯ=1\\ ϛ\neq Ѯ \end{array}}^{2}{\left(1-{\left(1-{ȿ}_{ϛԟ}\right)}^{r}{\left(1-{ȿ}_{Ѯᶅ}\right)}^{s}\right)}^{\frac{1}{2m\left(m-1\right)}}\right)}^{\frac{1}{r+s}} \end{array}\right)$$

*Using a similar argument for*m=2*we get*

$$PFS_{ft}BM^{r,s}\left({\hat{pf}}_{11},{\hat{pf}}_{12},.\;.\;.,{\hat{pf}}_{ͷ2}\right)={\left(\frac{1}{2ͷ\left(ͷ-1\right)}\begin{array}{c} \begin{array}{c} 2\\ \;⊕ \end{array}\\ \begin{array}{c} ԟ,ᶅ=1\\ ԟ\neq ᶅ \end{array} \end{array}\begin{array}{c} \begin{array}{c} ͷ\\ \;⊕ \end{array}\\ \begin{array}{c} ϛ,Ѯ=1\\ ϛ\neq Ѯ \end{array} \end{array}\left({\hat{pf}}_{ϛԟ}^{r}⨂{\hat{pf}}_{Ѯᶅ}^{s}\right)\right)}^{\frac{1}{r+s}}$$

$$=\left(\begin{array}{c} {\left(1-\left(\prod_{\begin{array}{c} ԟ,ᶅ=1\\ ԟ\neq ᶅ \end{array}}^{2}\prod_{\begin{array}{c} ϛ,Ѯ=1\\ ϛ\neq Ѯ \end{array}}^{ͷ}{\left(1-G_{ϛԟ}^{r}G_{Ѯᶅ}^{s}\right)}^{\frac{1}{2ͷ\left(ͷ-1\right)}}\right)\right)}^{\frac{1}{r+s}},1-{\left(1-\prod_{\begin{array}{c} ԟ,ᶅ=1\\ ԟ\neq ᶅ \end{array}}^{2}\prod_{\begin{array}{c} ϛ,Ѯ=1\\ ϛ\neq Ѯ \end{array}}^{ͷ}{\left(1-{\left(1-O_{ϛԟ}\right)}^{r}{\left(1-O_{Ѯᶅ}\right)}^{s}\right)}^{\frac{1}{2ͷ\left(ͷ-1\right)}}\right)}^{\frac{1}{r+s}},\\ 1-{\left(1-\prod_{\begin{array}{c} ԟ,ᶅ=1\\ ԟ\neq ᶅ \end{array}}^{2}\prod_{\begin{array}{c} ϛ,Ѯ=1\\ ϛ\neq Ѯ \end{array}}^{ͷ}{\left(1-{\left(1-{ȿ}_{ϛԟ}\right)}^{r}{\left(1-{ȿ}_{Ѯᶅ}\right)}^{s}\right)}^{\frac{1}{2ͷ\left(ͷ-1\right)}}\right)}^{\frac{1}{r+s}} \end{array}\right)$$

Thus the statement is true for ͷ=2 and m=2.

Step-2 *Now if* Eq. (5) *is valid for* $m={ԟ}_{1},ͷ={ԟ}_{2}+1$ and $m={ԟ}_{1}+1$, $ͷ={ԟ}_{2}$ *then for* $m={ԟ}_{1}+1$, $ͷ={ԟ}_{2}+1$,

$$\begin{array}{c} \begin{array}{c} {ԟ}_{1}+1\;⊕ \end{array}\\ \begin{array}{c} ԟ,ᶅ=1\\ ԟ\neq ᶅ \end{array} \end{array}\begin{array}{c} \begin{array}{c} {ԟ}_{2}+1\;⊕ \end{array}\\ \begin{array}{c} ϛ,Ѯ=1\\ ϛ\neq Ѯ \end{array} \end{array}\left({\hat{pf}}_{ϛԟ}^{r}⨂{\hat{pf}}_{Ѯᶅ}^{s}\right)=$$

(6) $$\left(\begin{array}{c} \begin{array}{c} {ԟ}_{1}\\ \;⊕ \end{array}\\ \begin{array}{c} ԟ,ᶅ=1\\ ԟ\neq ᶅ \end{array} \end{array}\begin{array}{c} \begin{array}{c} {ԟ}_{2}+1\\ \;⊕ \end{array}\\ \begin{array}{c} ϛ,Ѯ=1\\ ϛ\neq Ѯ \end{array} \end{array}\left({\hat{pf}}_{ϛԟ}^{r}⨂{\hat{pf}}_{Ѯᶅ}^{s}\right)\right)⊕\left(\begin{array}{c} \begin{array}{c} {ԟ}_{1}\\ \;⊕ \end{array}\\ ԟ=1 \end{array}\begin{array}{c} \begin{array}{c} {ԟ}_{2}+1\\ \;⊕ \end{array}\\ \begin{array}{c} ϛ,Ѯ=1\\ ϛ\neq Ѯ \end{array} \end{array}\left({\hat{pf}}_{ϛԟ}^{r}⨂{\hat{pf}}_{Ѯ\left({ԟ}_{1}+1\right)}^{s}\right)\right)⊕\left(\begin{array}{c} \begin{array}{c} {ԟ}_{1}\\ \;⊕ \end{array}\\ ԟ=1 \end{array}\begin{array}{c} \begin{array}{c} {ԟ}_{2}+1\\ \;⊕ \end{array}\\ \begin{array}{c} ϛ,Ѯ=1\\ ϛ\neq Ѯ \end{array} \end{array}\left({\hat{pf}}_{ϛ\left({ԟ}_{1}+1\right)}^{r}⨂{\hat{pf}}_{Ѯԟ}^{s}\right)\right)$$

*Now*

(7) $$\begin{array}{c} \begin{array}{c} {ԟ}_{2}+1\;⊕ \end{array}\\ \begin{array}{c} ϛ,Ѯ=1\\ ϛ\neq Ѯ \end{array} \end{array}\left({pf}_{ϛԟ}^{r}⨂{\hat{pf}}_{Ѯ\left({ԟ}_{1}+1\right)}^{s}\right)=\left(\begin{array}{c} 1-\prod_{\begin{array}{c} ϛ,Ѯ=1\\ ϛ\neq Ѯ \end{array}}^{{ԟ}_{2}+1}\left(1-G_{ϛԟ}^{r}G_{Ѯ\left({ԟ}_{1+1}\right)}^{s}\right),\prod_{\begin{array}{c} ϛ,Ѯ=1\\ ϛ\neq Ѯ \end{array}}^{{ԟ}_{2}+1}\left(1-{\left(1-O_{ϛԟ}\right)}^{r}{\left(1-O_{Ѯ\left({ԟ}_{1}+1\right)}\right)}^{s}\right),\\ \prod_{\begin{array}{c} ϛ,Ѯ=1\\ ϛ\neq Ѯ \end{array}}^{{ԟ}_{2}+1}\left(1-{\left(1-{ȿ}_{ϛԟ}\right)}^{r}{\left(1-{ȿ}_{Ѯ\left({ԟ}_{1}+1\right)}\right)}^{s}\right) \end{array}\right)$$

Using mathematical induction on ${ԟ}_{2}$.

(Step-2a) For ${ԟ}_{2}=1$.

$$\begin{array}{c} \begin{array}{c} 2\;⊕ \end{array}\\ \begin{array}{c} ϛ,Ѯ=1\\ ϛ\neq Ѯ \end{array} \end{array}\left({\hat{pf}}_{ϛԟ}^{r}⨂{\hat{pf}}_{Ѯ\left({ԟ}_{1}+1\right)}^{s}\right)=\left({\hat{pf}}_{1ԟ}^{r}⨂{\hat{pf}}_{2\left({ԟ}_{1}+1\right)}^{s}\right)⊕\left({\hat{pf}}_{2ԟ}^{r}⨂{\hat{pf}}_{1\left({ԟ}_{1}+1\right)}^{s}\right)$$

$$=\left(\begin{array}{c} 1-\prod_{\begin{array}{c} ϛ,Ѯ=1\\ ϛ\neq Ѯ \end{array}}^{2}\left(1-G_{ϛԟ}^{r}G_{Ѯ\left({ԟ}_{1+1}\right)}^{s}\right),\prod_{\begin{array}{c} ϛ,Ѯ=1\\ ϛ\neq Ѯ \end{array}}^{2}\left(1-{\left(1-O_{ϛԟ}\right)}^{r}{\left(1-O_{Ѯ\left({ԟ}_{1}+1\right)}\right)}^{s}\right),\\ \prod_{\begin{array}{c} ϛ,Ѯ=1\\ ϛ\neq Ѯ \end{array}}^{2}\left(1-{\left(1-{ȿ}_{ϛԟ}\right)}^{r}\times {\left(1-{ȿ}_{Ѯ\left({ԟ}_{1}+1\right)}\right)}^{s}\right) \end{array}\right)$$

(Step 2b) Assume the result is valid for ${ԟ}_{2}=z\text{,}$.

$$\begin{array}{c} \begin{array}{c} z+1\;⊕ \end{array}\\ \begin{array}{c} ϛ,Ѯ=1\\ ϛ\neq Ѯ \end{array} \end{array}\left({\hat{pf}}_{ϛԟ}^{r}⨂{\hat{pf}}_{Ѯ\left({ԟ}_{1}+1\right)}^{s}\right)=\left(\begin{array}{c} 1-\prod_{\begin{array}{c} ϛ,Ѯ=1\\ ϛ\neq Ѯ \end{array}}^{z+1}\left(1-G_{ϛԟ}^{r}G_{Ѯ\left({ԟ}_{1+1}\right)}^{s}\right),\prod_{\begin{array}{c} ϛ,Ѯ=1\\ ϛ\neq Ѯ \end{array}}^{z+1}\left(1-{\left(1-O_{ϛԟ}\right)}^{r}\times {\left(1-O_{Ѯ\left({ԟ}_{1}+1\right)}\right)}^{s}\right),\\ \prod_{\begin{array}{c} ϛ,Ѯ=1\\ ϛ\neq Ѯ \end{array}}^{z+1}\left(1-{\left(1-{ȿ}_{ϛԟ}\right)}^{r}\times {\left(1-{ȿ}_{Ѯ\left({ԟ}_{1}+1\right)}\right)}^{s}\right) \end{array}\right)$$

*Then for*${ԟ}_{2}=z+1$, *we get*

$$\begin{array}{c} \begin{array}{c} z+2\;⊕ \end{array}\\ \begin{array}{c} ϛ,Ѯ=1\\ ϛ\neq Ѯ \end{array} \end{array}\left({\hat{pf}}_{ϛԟ}^{r}⨂{\hat{pf}}_{Ѯ\left({ԟ}_{1}+1\right)}^{s}\right)$$

$$=\left(\begin{array}{c} \begin{array}{c} z+1\\ \;⊕ \end{array}\\ \begin{array}{c} ϛ,Ѯ=1\\ ϛ\neq Ѯ \end{array} \end{array}\left({\hat{pf}}_{ϛԟ}^{r}⨂{\hat{pf}}_{Ѯ\left({ԟ}_{1}+1\right)}^{s}\right)\right)⊕\left(\begin{array}{c} \begin{array}{c} z+1\\ \;⊕ \end{array}\\ ϛ=1 \end{array}\left({\hat{pf}}_{\left(z+2\right)ԟ}^{r}⨂{\hat{pf}}_{ϛ\left({ԟ}_{1}+1\right)}^{s}\right)\right)⊕\left(\begin{array}{c} \begin{array}{c} z+1\\ \;⊕ \end{array}\\ ϛ=1 \end{array}\left({\hat{pf}}_{ϛԟ}^{r}⨂{\hat{ơ}}_{\left(z+2\right)\left(ԟ+1\right)}^{s}\right)\right)$$

$$=\left(\begin{array}{c} 1-\prod_{\begin{array}{c} ϛ,Ѯ=1\\ ϛ\neq Ѯ \end{array}}^{z+1}\left(1-G_{ϛԟ}^{r}G_{Ѯ\left({ԟ}_{1+1}\right)}^{s}\right),\prod_{\begin{array}{c} ϛ,Ѯ=1\\ ϛ\neq Ѯ \end{array}}^{z+1}\left(1-{\left(1-O_{ϛԟ}\right)}^{r}{\left(1-O_{Ѯ\left({ԟ}_{1}+1\right)}\right)}^{s}\right),\\ \prod_{\begin{array}{c} ϛ,Ѯ=1\\ ϛ\neq Ѯ \end{array}}^{z+1}\left(1-{\left(1-{ȿ}_{ϛԟ}\right)}^{r}{\left(1-{ȿ}_{Ѯ\left({ԟ}_{1}+1\right)}\right)}^{s}\right) \end{array}\right)⊕$$

$$\left(\begin{array}{c} 1-\prod_{ϛ=1}^{z+1}\left(1-G_{\left(z+2\right)ԟ}^{r}G_{ϛ\left({ԟ}_{1+1}\right)}^{s}\right),\prod_{ϛ=1}^{z+1}\left(1-{\left(1-O_{\left(z+2\right)ԟ}\right)}^{r}{\left(1-O_{ϛ\left({ԟ}_{1}+1\right)}\right)}^{s}\right),\\ \prod_{ϛ=1}^{z+1}\left(1-{\left(1-{ȿ}_{\left(z+2\right)ԟ}\right)}^{r}{\left(1-{ȿ}_{ϛ\left({ԟ}_{1}+1\right)}\right)}^{s}\right) \end{array}\right)⊕$$

$$\left(\begin{array}{c} 1-\prod_{ϛ=1}^{z+1}\left(G_{ϛԟ}^{r}G_{\left(z+2\right)\left({ԟ}_{1+1}\right)}^{s}\right),\prod_{ϛ=1}^{z+1}\left(1-{\left(1-O_{ϛԟ}\right)}^{r}{\left(1-O_{\left(z+2\right)\left({ԟ}_{1}+1\right)}\right)}^{s}\right)\text{,}\\ \prod_{ϛ=1}^{z+1}\left(1-{\left(1-{ȿ}_{ϛԟ}\right)}^{r}{\left(1-{ȿ}_{\left(z+2\right)\left({ԟ}_{1}+1\right)}\right)}^{s}\right) \end{array}\right)$$

$$=\left(\begin{array}{c} 1-\prod_{\begin{array}{c} ϛ,Ѯ=1\\ ϛ\neq Ѯ \end{array}}^{z+2}\left(1-G_{ϛԟ}^{r}G_{Ѯ\left({ԟ}_{1+1}\right)}^{s}\right),\prod_{\begin{array}{c} ϛ,Ѯ=1\\ ϛ\neq Ѯ \end{array}}^{z+2}\left(1-{\left(1-O_{ϛԟ}\right)}^{r}{\left(1-O_{Ѯ\left({ԟ}_{1}+1\right)}\right)}^{s}\right),\\ \prod_{\begin{array}{c} ϛ,Ѯ=1\\ ϛ\neq Ѯ \end{array}}^{z+2}\left(1-{\left(1-{ȿ}_{ϛԟ}\right)}^{r}{\left(1-{ȿ}_{Ѯ\left({ԟ}_{1}+1\right)}\right)}^{s}\right) \end{array}\right)$$

*Hence* Eq. (7) *is valid for* ${ԟ}_{2}=z+1$. *Hence*, *we get*

$$\begin{array}{c} \begin{array}{c} {ԟ}_{1}\;⊕ \end{array}\\ ԟ=1 \end{array}\begin{array}{c} \begin{array}{c} {ԟ}_{2}+1\;⊕ \end{array}\\ \begin{array}{c} ϛ,Ѯ=1\\ ϛ\neq Ѯ \end{array} \end{array}\left({\hat{pf}}_{ϛԟ}^{r}⨂{\hat{pf}}_{Ѯ\left({ԟ}_{1}+1\right)}^{s}\right)$$

$$=\left(\begin{array}{c} 1-\prod_{ԟ=1}^{{ԟ}_{1}}\prod_{\begin{array}{c} ϛ,Ѯ=1\\ ϛ\neq Ѯ \end{array}}^{{ԟ}_{2}+1}\left(1-G_{ϛԟ}^{r}G_{Ѯ\left({ԟ}_{1}+1\right)}^{s}\right),\prod_{ԟ=1}^{{ԟ}_{1}}\prod_{\begin{array}{c} ϛ,Ѯ=1\\ ϛ\neq Ѯ \end{array}}^{{ԟ}_{2}+1}\left(1-{\left(1-O_{ϛԟ}\right)}^{r}{\left(1-O_{Ѯ\left({ԟ}_{1}+1\right)}\right)}^{s}\right),\\ \prod_{ԟ=1}^{{ԟ}_{1}}\prod_{\begin{array}{c} ϛ,Ѯ=1\\ ϛ\neq Ѯ \end{array}}^{{ԟ}_{2}+1}\left(1-{\left(1-{ȿ}_{ϛԟ}\right)}^{r}{\left(1-{ȿ}_{Ѯ\left({ԟ}_{1}+1\right)}\right)}^{s}\right) \end{array}\right)$$

*Similarly*, *we can show that*

$$\begin{array}{c} \begin{array}{c} {ԟ}_{1}\;⊕ \end{array}\\ ԟ=1 \end{array}\begin{array}{c} \begin{array}{c} {ԟ}_{2}+1\;⊕ \end{array}\\ \begin{array}{c} ϛ,Ѯ=1\\ ϛ\neq Ѯ \end{array} \end{array}\left({\hat{pf}}_{ϛ\left({ԟ}_{1}+1\right)}^{r}⨂{pf}_{Ѯԟ}^{s}\right)$$

$$=\left(\begin{array}{c} 1-\prod_{ԟ=1}^{{ԟ}_{1}}\prod_{\begin{array}{c} ϛ,Ѯ=1\\ ϛ\neq Ѯ \end{array}}^{{ԟ}_{2}+1}\left(1-G_{ϛ\left({ԟ}_{1}+1\right)}^{r}G_{Ѯԟ}^{s}\right),\prod_{ԟ=1}^{{ԟ}_{1}}\prod_{\begin{array}{c} ϛ,Ѯ=1\\ ϛ\neq Ѯ \end{array}}^{{ԟ}_{2}+1}\left(1-{\left(1-O_{ϛ\left({ԟ}_{1}+1\right)}\right)}^{r}{\left(1-O_{Ѯԟ}\right)}^{s}\right),\\ \prod_{ԟ=1}^{{ԟ}_{1}}\prod_{\begin{array}{c} ϛ,Ѯ=1\\ ϛ\neq Ѯ \end{array}}^{{ԟ}_{2}+1}\left(1-{\left(1-{ȿ}_{ϛ\left({ԟ}_{1}+1\right)}\right)}^{r}{\left(1-{ȿ}_{Ѯԟ}\right)}^{s}\right) \end{array}\right)\left(8\right)$$

*From* Eqs. (6), (7) *we have*

$$\begin{array}{c} \begin{array}{c} {ԟ}_{1}+1\;⊕ \end{array}\\ \begin{array}{c} ԟ,ᶅ=1\\ ԟ\neq ᶅ \end{array} \end{array}\begin{array}{c} \begin{array}{c} {ԟ}_{2}+1\;⊕ \end{array}\\ \begin{array}{c} ϛ,Ѯ=1\\ ϛ\neq Ѯ \end{array} \end{array}\left({\hat{pf}}_{ϛԟ}^{r}⨂{\hat{pf}}_{Ѯᶅ}^{s}\right)=\left(\begin{array}{c} 1-\prod_{ԟ=1}^{{ԟ}_{1}}\prod_{\begin{array}{c} ϛ,Ѯ=1\\ ϛ\neq Ѯ \end{array}}^{{ԟ}_{2}+1}\left(1-G_{ϛԟ}^{r}G_{Ѯᶅ}^{s}\right),\prod_{\begin{array}{c} ԟ,ᶅ=1\\ ԟ\neq ᶅ \end{array}}^{{ԟ}_{1}}\prod_{\begin{array}{c} ϛ,Ѯ=1\\ ϛ\neq Ѯ \end{array}}^{{ԟ}_{2}+1}\left(1-{\left(1-O_{ϛԟ}\right)}^{r}{\left(1-O_{Ѯᶅ}\right)}^{s}\right),\\ \prod_{\begin{array}{c} ԟ,ᶅ=1\\ ԟ\neq ᶅ \end{array}}^{{ԟ}_{1}}\prod_{\begin{array}{c} ϛ,Ѯ=1\\ ϛ\neq Ѯ \end{array}}^{{ԟ}_{2}+1}\left(1-{\left(1-{ȿ}_{ϛԟ}\right)}^{r}{\left(1-{ȿ}_{Ѯᶅ}\right)}^{s}\right) \end{array}\right)⊕$$

$$\left(\begin{array}{c} 1-\prod_{ԟ=1}^{{ԟ}_{1}}\prod_{\begin{array}{c} ϛ,Ѯ=1\\ ϛ\neq Ѯ \end{array}}^{{ԟ}_{2}+1}\left(1-G_{ϛԟ}^{r}G_{Ѯ\left(\left({ԟ}_{1}+1\right)\right)}^{s}\right),\prod_{ԟ=1}^{{ԟ}_{1}}\prod_{\begin{array}{c} ϛ,Ѯ=1\\ ϛ\neq Ѯ \end{array}}^{{ԟ}_{2}+1}\left(1-{\left(1-O_{ϛԟ}\right)}^{r}{\left(1-O_{Ѯ\left({ԟ}_{1}+1\right)}\right)}^{s}\right),\\ \prod_{ԟ=1}^{{ԟ}_{1}}\prod_{\begin{array}{c} ϛ,Ѯ=1\\ ϛ\neq Ѯ \end{array}}^{{ԟ}_{2}+1}\left(1-{\left(1-{ȿ}_{ϛԟ}\right)}^{r}{\left(1-{ȿ}_{Ѯ\left({ԟ}_{1}+1\right)}\right)}^{s}\right) \end{array}\right)⊕$$

$$\left(\begin{array}{c} 1-\prod_{ԟ=1}^{{ԟ}_{1}}\prod_{\begin{array}{c} ϛ,Ѯ=1\\ ϛ\neq Ѯ \end{array}}^{{ԟ}_{2}+1}\left(1-G_{ϛ\left({ԟ}_{1}+1\right)}^{r}G_{Ѯԟ}^{s}\right),\prod_{ԟ=1}^{{ԟ}_{1}}\prod_{\begin{array}{c} ϛ,Ѯ=1\\ ϛ\neq Ѯ \end{array}}^{{ԟ}_{2}+1}\left(1-{\left(1-O_{ϛ\left({ԟ}_{1}+1\right)}\right)}^{r}{\left(1-O_{Ѯԟ}\right)}^{s}\right),\\ \prod_{ԟ=1}^{{ԟ}_{1}}\prod_{\begin{array}{c} ϛ,Ѯ=1\\ ϛ\neq Ѯ \end{array}}^{{ԟ}_{2}+1}\left(1-{\left(1-{ȿ}_{ϛ\left({ԟ}_{1}+1\right)}\right)}^{r}{\left(1-{ȿ}_{Ѯԟ}\right)}^{s}\right) \end{array}\right)$$

$$=\left(\begin{array}{c} 1-\prod_{\begin{array}{c} ԟ,ᶅ=1\\ ԟ\neq ᶅ \end{array}}^{{ԟ}_{1}+1}\prod_{\begin{array}{c} ϛ,Ѯ=1\\ ϛ\neq Ѯ \end{array}}^{{ԟ}_{2}+1}\left(1-G_{ϛԟ}^{r}G_{Ѯᶅ}^{s}\right),\prod_{\begin{array}{c} ԟ,ᶅ=1\\ ԟ\neq ᶅ \end{array}}^{{ԟ}_{1}+1}\prod_{\begin{array}{c} ϛ,Ѯ=1\\ ϛ\neq Ѯ \end{array}}^{{ԟ}_{2}+1}\left(1-{\left(1-O_{ϛԟ}\right)}^{r}{\left(1-O_{Ѯᶅ}\right)}^{s}\right),\\ \prod_{\begin{array}{c} ԟ,ᶅ=1\\ ԟ\neq ᶅ \end{array}}^{{ԟ}_{1}+1}\prod_{\begin{array}{c} ϛ,Ѯ=1\\ ϛ\neq Ѯ \end{array}}^{{ԟ}_{2}+1}\left(1-{\left(1-{ȿ}_{ϛԟ}\right)}^{r}{\left(1-{ȿ}_{Ѯᶅ}\right)}^{s}\right) \end{array}\right)\text{.}$$

So, from the above expression, it is clear that Eq. (5) is valid for all positive integers m,ͷ..

Example 1 Assume that Mr. X wants to select the best car brand. Also, assume that $\delta =\left\{\delta_{1},\delta_{2},\delta_{3},\delta_{4}\right\}$ is the set of experts that describe the attractiveness of the best car brand corresponding to a set of parameters given as $\bar{ͳ}=\left\{\varepsilon_{1}=Price,\varepsilon_{2}=Upgrade\;Model,\varepsilon_{3}=Features\right\}\text{.}$ Let experts express their assessment in the shape of $PFS_{ft}Ns$ as given in Table 1.

**Table 1:** Picture fuzzy soft information.

	$\varepsilon_{1}$	$\varepsilon_{2}$	$\varepsilon_{3}$
$\delta_{1}$	(0.2,0.3,0.4)	(0.2,0.4,0.3)	(0.1,0.4,0.3)
$\delta_{2}$	(0.5,0.1,0.2)	(0.1,0.1,0.5)	(0.3,0.1,0.2)
$\delta_{3}$	(0.3,0.3,0.1)	(0.2,0.3,0.2)	(0.2,0.1,0.1)
$\delta_{4}$	(0.4,0.1,0.3)	(0.4,0.4,0.1)	(0.5,0.2,0.1)

*For convenience assume that*r,s=1*be two real numbers then we get*

$${\left(1-\left(\prod_{\begin{array}{c} ԟ,ᶅ=1\\ ԟ\neq ᶅ \end{array}}^{3}\prod_{\begin{array}{c} ϛ,Ѯ=1\\ ϛ\neq Ѯ \end{array}}^{4}{\left(1-G_{ϛԟ}^{r}G_{Ѯᶅ}^{s}\right)}^{\frac{1}{34\left(3-1\right)\left(4-1\right)}}\right)\right)}^{\frac{1}{r+s}}$$

$$={\left(1-\left(\begin{array}{c} {\left(1-\left(0.2\right)\left(0.1\right)\right)}^{\frac{1}{72}}\times {\left(1-\left(0.2\right)\left(0.2\right)\right)}^{\frac{1}{72}}\times {\left(1-\left(0.2\right)\left(0.4\right)\right)}^{\frac{1}{72}}\ldots \\ \times {\left(1-\left(0.5\right)\left(0.2\right)\right)}^{\frac{1}{72}}\times {\left(1-\left(0.5\right)\left(0.1\right)\right)}^{\frac{1}{72}}\times {\left(1-\left(0.5\right)\left(0.2\right)\right)}^{\frac{1}{72}} \end{array}\right)\right)}^{\frac{1}{2}}$$

=(0.2095)

*And*

$$\left(1-{\left(1-\prod_{\begin{array}{c} ԟ,ᶅ=1\\ ԟ\neq ᶅ \end{array}}^{m}\prod_{\begin{array}{c} ϛ,Ѯ=1\\ ϛ\neq Ѯ \end{array}}^{ͷ}{\left(1-{\left(1-O_{ϛԟ}\right)}^{r}{\left(1-O_{Ѯᶅ}\right)}^{s}\right)}^{\frac{1}{mͷ\left(m-1\right)\left(ͷ-1\right)}}\right)}^{\frac{1}{r+s}}\right)$$

$$=\left(1-{\left(1-{\left(1-\left(1-0.3\right)\left(1-0.1\right)\right)}^{\frac{1}{72}}\times {\left(1-\left(1-0.3\right)\left(1-0.3\right)\right)}^{\frac{1}{72}}\times {\left(1-\left(1-0.3\right)\left(1-0.4\right)\right)}^{\frac{1}{72}}\ldots \times {\left(1-\left(1-0.2\right)\left(1-0.4\right)\right)}^{\frac{1}{72}}\times {\left(1-\left(1-0.2\right)\left(1-0.1\right)\right)}^{\frac{1}{72}}\times {\left(1-\left(1-0.3\right)\left(1-0.3\right)\right)}^{\frac{1}{72}}\right)}^{\frac{1}{2}}\right)$$

=(0.3144)

*Similarly*

$$\left(1-{\left(1-\prod_{\begin{array}{c} ԟ,ᶅ=1\\ ԟ\neq ᶅ \end{array}}^{m}\prod_{\begin{array}{c} ϛ,Ѯ=1\\ ϛ\neq Ѯ \end{array}}^{ͷ}{\left(1-{\left(1-{ȿ}_{ϛԟ}\right)}^{r}{\left(1-{ȿ}_{Ѯᶅ}\right)}^{s}\right)}^{\frac{1}{mͷ\left(m-1\right)\left(ͷ-1\right)}}\right)}^{\frac{1}{r+s}}\right)$$

$$=\left(1-{\left(1-{\left(1-\left(1-0.4\right)\left(1-0.5\right)\right)}^{\frac{1}{72}}\times {\left(1-\left(1-0.4\right)\left(1-0.2\right)\right)}^{\frac{1}{72}}\times {\left(1-\left(1-0.4\right)\left(1-0.1\right)\right)}^{\frac{1}{72}}\ldots \times {\left(1-\left(1-0.1\right)\left(1-0.3\right)\right)}^{\frac{1}{72}}\times {\left(1-\left(1-0.1\right)\left(1-0.5\right)\right)}^{\frac{1}{72}}\times {\left(1-\left(1-0.1\right)\left(1-0.2\right)\right)}^{\frac{1}{72}}\right)}^{\frac{1}{2}}\right)$$

=(0.3400)

Hence $PFS_{ft}BM^{1,1}\left({\hat{pf}}_{11},{\hat{pf}}_{12},.\;.\;.,{\hat{pf}}_{43}\right)=\left(0.2095,0.3144,0.3400\right)$.

*Further*, *we will discuss that*$PFS_{ft}BM$*operator has the following properties*

1. **(Idempotency)** If ${\hat{pf}}_{ϛԟ}=\hat{pf}=\left\langle G,O,ȿ\right\rangle$ for all ϛ,ԟ then $PFSBM^{r,s}\left({\hat{pf}}_{11},{\hat{pf}}_{12},.\;.\;.,{\hat{pf}}_{ͷm}\right)=\hat{pf}\text{.}$.
2. **(*Boundedness*)** Let ${\hat{pf}}^{-}=\left\langle \min_{ԟ}\min_{ϛ}\left\{G_{ϛԟ}\right\},\max_{ԟ}\max_{ϛ}\left\{O_{ϛԟ}\right\},\max_{ԟ}\max_{ϛ}\left\{{ȿ}_{ϛԟ}\right\}\right\rangle$ *and*

${pf}^{+}=\left\langle \max_{ԟ}\max_{ϛ}\left\{G_{ϛԟ}\right\},\min_{ԟ}\min_{ϛ}\left\{O_{ϛԟ}\right\},\min_{ԟ}\min_{ϛ}\left\{{ȿ}_{ϛԟ}\right\}\right\rangle$ then

${\hat{pf}}^{-}\leq PFSBM^{r,s}\left({\hat{pf}}_{11},{\hat{pf}}_{12},.\;.\;.,{\hat{pf}}_{ͷm}\right)\leq {\hat{pf}}^{+}\text{.}$.

3. **(*Monotonicity*)***Let*${\hat{pf}}_{ϛԟ}^{\prime }$*be another family of*$PFS_{ft}Ns$*such that*${\hat{pf}}_{ϛԟ}\leq {\hat{pf}}_{ϛԟ}^{\prime }$*for all*ԟ,ϛ,*then*

$PFS_{ft}BM^{r,s}\left({\hat{pf}}_{11},{pf}_{12},.\;.\;.,{\hat{pf}}_{ͷm}\right)\leq PFS_{ft}BM^{r,s}\left({\hat{pf}}_{11}^{\prime },{\hat{pf}}_{12}^{\prime },.\;.\;.,{\hat{pf}}_{ͷm}^{\prime }\right)\text{.}$.

4. **(*Commutativity*)**${\hat{pf}}_{ϛԟ}\left(ϛ=1,2,.\;.\;.\;.,ͷ;ԟ=1,2,.\;.\;.\;.m\right)$*be a family of*$PFS_{ft}Ns\text{,}$*then*

$PFS_{ft}BM^{r,s}\left({\hat{pf}}_{11},{\hat{pf}}_{12},.\;.\;.,{\hat{pf}}_{ͷm}\right)=PFS_{ft}BM^{r,s}\left({\hat{pf}}_{11}^{⋆},{\hat{pf}}_{12}^{⋆},.\;.\;.,{\hat{pf}}_{ͷm}^{⋆}\right)\text{.}$.

Where $\left({\hat{pf}}_{11}^{⋆},{\hat{pf}}_{12}^{⋆},.\;.\;.,{\hat{pf}}_{ͷm}^{⋆}\right)$ is any permutation of $\left({\hat{pf}}_{11},{\hat{pf}}_{12},.\;.\;.,{\hat{pf}}_{ͷm}\right)\text{.}$.

## 4 Weighted picture fuzzy soft Bonferroni mean $\left(WPFS_{ft}BM\right)$ operator

In this part of the article, we will define $WPFS_{ft}BM$ operator to consider the weights of both experts and parameters so that more close results can be obtained for the desired goal.

Definition 8 For a family of $PFS_{ft}Ns$, a $WPFS_{ft}BM$ operator is a function $WPFS_{ft}BM:z^{ͷ}⟶z$ defined by

$$WPFS_{ft}BM^{r,s}\left({\hat{pf}}_{11},{\hat{pf}}_{12},.\;.\;.,{\hat{pf}}_{ͷm}\right)$$

(9) $$={\left(\frac{1}{mͷ\left(m-1\right)\left(ͷ-1\right)}\begin{array}{c} \begin{array}{c} m\\ \;⊕ \end{array}\\ \begin{array}{c} ԟ,ᶅ=1\\ ԟ\neq ᶅ \end{array} \end{array}\begin{array}{c} \begin{array}{c} ͷ\\ \;⊕ \end{array}\\ \begin{array}{c} ϛ,Ѯ=1\\ ϛ\neq Ѯ \end{array} \end{array}{\left(o_{ԟ}\left(f_{ϛ}{\hat{pf}}_{ϛԟ}\right)\right)}^{r}{⨂\left(o_{ᶅ}\left(f_{ϛ}{\hat{pf}}_{Ѯᶅ}\right)\right)}^{s}\right)}^{\frac{1}{r+s}}$$

Where $o={\left(o_{1},o_{2},.\;.\;.\;.o_{m}\right)}^{T}$ and $f={\left(f_{1},f_{2},.\;.\;.\;.,f_{ͷ}\right)}^{T}$ be the weight vectors (WVs) of parameters and the experts respectively with constraints that $\sum_{ϛ=1}^{ͷ}o_{ԟ}=1\text{,}$ and $\sum_{ϛ=1}^{ͷ}f_{ϛ}=1$.

Now using equation (9), we can define $WPFS_{ft}BM$ operators as follows

Theorem 2 *For the family of*$PFS_{ft}Ns$, *the aggregated results using*$WPFS_{ft}BM$*operator is again a*$PFS_{ft}N$*defined by*

$$WPFS_{ft}BM^{r,s}\left({\hat{pf}}_{11},{\hat{pf}}_{12},.\;.\;.,{\hat{pf}}_{ͷm}\right)$$

(10) $$=\left(\begin{array}{c} {\left(1-\left(\prod_{\begin{array}{c} ԟ,ᶅ=1\\ ԟ\neq ᶅ \end{array}}^{m}\prod_{\begin{array}{c} ϛ,Ѯ=1\\ ϛ\neq Ѯ \end{array}}^{ͷ}{\left({1-\left(1-{\left(1-G_{ϛԟ}\right)}^{o_{ԟ}f_{ϛ}}\right)}^{r}{\left(1-{\left(1-G_{Ѯᶅ}\right)}^{{oq}_{ᶅ}f_{Ѯ}}\right)}^{s}\right)}^{\frac{1}{mͷ\left(m-1\right)\left(ͷ-1\right)}}\right)\right)}^{\frac{1}{r+s}},\\ 1-{\left(1-\prod_{\begin{array}{c} ԟ,ᶅ=1\\ ԟ\neq ᶅ \end{array}}^{m}\prod_{\begin{array}{c} ϛ,Ѯ=1\\ ϛ\neq Ѯ \end{array}}^{ͷ}{\left(1-{\left(1-O_{ϛԟ}^{o_{ԟ}f_{ϛ}}\right)}^{r}{\left(1-O_{Ѯᶅ}^{o_{ᶅ}f_{Ѯ}}\right)}^{s}\right)}^{\frac{1}{mͷ\left(m-1\right)\left(ͷ-1\right)}}\right)}^{\frac{1}{r+s}},\\ 1-{\left(1-\prod_{\begin{array}{c} ԟ,ᶅ=1\\ ԟ\neq ᶅ \end{array}}^{m}\prod_{\begin{array}{c} ϛ,Ѯ=1\\ ϛ\neq Ѯ \end{array}}^{ͷ}{\left(1-{\left(1-{ȿ}_{ϛԟ}^{{oq}_{ԟ}f_{ϛ}}\right)}^{r}{\left(1-{ȿ}_{Ѯᶅ}^{o_{ᶅ}f_{Ѯ}}\right)}^{s}\right)}^{\frac{1}{mͷ\left(m-1\right)\left(ͷ-1\right)}}\right)}^{\frac{1}{r+s}} \end{array}\right)$$

Example 2 *Using the data of*example 1*and suppose that WVs for experts and parameters are given respectively as*{0.24,0.34,0.42}*and*{0.20,0.30,0.32,0.18}. *Now we use the data in*Table 1*and* equation (10) *to find the value of the* $WPFS_{ft}BM$ *operator*.

$$WPFS_{ft}BM^{r,s}\left({\hat{pf}}_{11},{\hat{pf}}_{12},.\;.\;.,{\hat{pf}}_{ͷm}\right)=\left(0.6661,0.05717,0.031198\right)$$

## 5 Special cases of the initiated work

Here in this part, we will propose the special cases for introduced work to show the effectiveness and contribution of initiated work.

Case 1 If s⟶0, then the defined operators turn down to weighted $PFS_{ft}$ mean operators are given by:

$$\underset{s⟶0}{\mathrm{Lim}}WPFS_{ft}BM^{r,s}\left({\hat{pf}}_{11},{\hat{pf}}_{12},.\;.\;.,{\hat{pf}}_{ͷm}\right)$$

$$=\lim_{s⟶0}\left(\begin{array}{c} {\left(1-\left(\prod_{\begin{array}{c} ԟ,ᶅ=1\\ ԟ\neq ᶅ \end{array}}^{m}\prod_{\begin{array}{c} ϛ,Ѯ=1\\ ϛ\neq Ѯ \end{array}}^{ͷ}{\left({1-\left(1-{\left(1-G_{ϛԟ}\right)}^{o_{ԟ}f_{ϛ}}\right)}^{r}{\left(1-{\left(1-G_{Ѯᶅ}\right)}^{o_{ᶅ}f_{Ѯ}}\right)}^{s}\right)}^{\frac{1}{mͷ\left(m-1\right)\left(ͷ-1\right)}}\right)\right)}^{\frac{1}{r+s}},\\ 1-{\left(1-\prod_{\begin{array}{c} ԟ,ᶅ=1\\ ԟ\neq ᶅ \end{array}}^{m}\prod_{\begin{array}{c} ϛ,Ѯ=1\\ ϛ\neq Ѯ \end{array}}^{ͷ}{\left(1-{\left(1-O_{ϛԟ}^{o_{ԟ}f_{ϛ}}\right)}^{r}{\left(1-O_{Ѯᶅ}^{o_{ᶅ}f_{Ѯ}}\right)}^{s}\right)}^{\frac{1}{mͷ\left(m-1\right)\left(ͷ-1\right)}}\right)}^{\frac{1}{r+s}},\\ 1-{\left(1-\prod_{\begin{array}{c} ԟ,ᶅ=1\\ ԟ\neq ᶅ \end{array}}^{m}\prod_{\begin{array}{c} ϛ,Ѯ=1\\ ϛ\neq Ѯ \end{array}}^{ͷ}{\left(1-{\left(1-{ȿ}_{ϛԟ}^{o_{ԟ}f_{ϛ}}\right)}^{r}{\left(1-{ȿ}_{Ѯᶅ}^{o_{ᶅ}f_{Ѯ}}\right)}^{s}\right)}^{\frac{1}{mͷ\left(m-1\right)\left(ͷ-1\right)}}\right)}^{\frac{1}{r+s}} \end{array}\right)$$

$$=\left(\begin{array}{c} {\left(1-\left(\prod_{ԟ=1}^{m}\prod_{ϛ=1}^{ͷ}{\left({1-\left(1-{\left(1-G_{ϛԟ}\right)}^{o_{ԟ}f_{ϛ}}\right)}^{r}\right)}^{\frac{\left(m-1\right)\left(ͷ-1\right)}{mͷ\left(m-1\right)\left(ͷ-1\right)}}\right)\right)}^{\frac{1}{r}},\\ 1-{\left(1-\prod_{ԟ=1}^{m}\prod_{ϛ=1}^{ͷ}{\left(1-{\left(1-O_{ϛԟ}^{o_{ԟ}f_{ϛ}}\right)}^{r}\right)}^{\frac{\left(m-1\right)\left(ͷ-1\right)}{mͷ\left(m-1\right)\left(ͷ-1\right)}}\right)}^{\frac{1}{r}},\\ 1-{\left(1-\prod_{ԟ=1}^{m}\prod_{ϛ=1}^{ͷ}{\left(1-{\left(1-{ȿ}_{ϛԟ}^{o_{ԟ}f_{ϛ}}\right)}^{r}\right)}^{\frac{\left(m-1\right)\left(ͷ-1\right)}{mͷ\left(m-1\right)\left(ͷ-1\right)}}\right)}^{\frac{1}{r}} \end{array}\right)$$

$$=\left(\begin{array}{c} {\left(1-\left(\prod_{ԟ=1}^{m}\prod_{ϛ=1}^{ͷ}{\left({1-\left(1-{\left(1-G_{ϛԟ}\right)}^{o_{ԟ}f_{ϛ}}\right)}^{r}\right)}^{\frac{1}{mͷ}}\right)\right)}^{\frac{1}{r}},\\ 1-{\left(1-\prod_{ԟ=1}^{m}\prod_{ϛ=1}^{ͷ}{\left(1-{\left(1-O_{ϛԟ}^{{oq}_{ԟ}f_{ϛ}}\right)}^{r}\right)}^{\frac{1}{mͷ}}\right)}^{\frac{1}{r}},\\ 1-{\left(1-\prod_{ԟ=1}^{m}\prod_{ϛ=1}^{ͷ}{\left(1-{\left(1-{ȿ}_{ϛԟ}^{{oq}_{ԟ}f_{ϛ}}\right)}^{r}\right)}^{\frac{1}{mͷ}}\right)}^{\frac{1}{r}} \end{array}\right)$$

$${=\left(\frac{1}{mͷ}\begin{array}{c} \begin{array}{c} m\\ \;⊕ \end{array}\\ ԟ=1 \end{array}\begin{array}{c} \begin{array}{c} ͷ\\ \;⊕ \end{array}\\ ϛ=1 \end{array}{\left(o_{ԟ}\left(f_{ϛ}\hat{pf_{ϛԟ}}\right)\right)}^{r}\right)}^{\frac{1}{r}}$$

$$=WPFS_{ft}BM^{r,0}\left({\hat{pf}}_{11},{\hat{pf}}_{12},.\;.\;.,{\hat{pf}}_{ͷm}\right)$$

Case 2 If r=2 and s⟶0, then the initiated operator degenerate into weighted $PFS_{ft}$ square mean operators are given by

$$WPFS_{ft}BM^{2,0}\left({\hat{pf}}_{11},{\hat{pf}}_{12},.\;.\;.,{\hat{pf}}_{ͷm}\right)$$

$$=\left(\begin{array}{c} {\left(1-\left(\prod_{ԟ=1}^{m}\prod_{ϛ=1}^{ͷ}{\left({1-\left(1-{\left(1-G_{ϛԟ}\right)}^{o_{ԟ}f_{ϛ}}\right)}^{2}\right)}^{\frac{1}{mͷ}}\right)\right)}^{\frac{1}{2}},\\ 1-{\left(1-\prod_{ԟ=1}^{m}\prod_{ϛ=1}^{ͷ}{\left(1-{\left(1-O_{ϛԟ}^{o_{ԟ}f_{ϛ}}\right)}^{2}\right)}^{\frac{1}{mͷ}}\right)}^{\frac{1}{2}},\\ 1-{\left(1-\prod_{ԟ=1}^{m}\prod_{ϛ=1}^{ͷ}{\left(1-{\left(1-{ȿ}_{ϛԟ}^{{oq}_{ԟ}f_{ϛ}}\right)}^{2}\right)}^{\frac{1}{mͷ}}\right)}^{\frac{1}{2}} \end{array}\right)$$

$${=\left(\frac{1}{mͷ}\begin{array}{c} \begin{array}{c} m\\ \;⊕ \end{array}\\ ԟ=1 \end{array}\begin{array}{c} \begin{array}{c} ͷ\\ \;⊕ \end{array}\\ ϛ=1 \end{array}{\left(o_{ԟ}\left(f_{ϛ}\hat{pf_{ϛԟ}}\right)\right)}^{2}\right)}^{\frac{1}{2}}$$

Case 3 If r=1 and s⟶0, then the initiated operator degenerate into weighted $PFS_{ft}$ average operators are given by

$$WPFS_{ft}BM^{1,0}\left({\hat{pf}}_{11},{\hat{pf}}_{12},.\;.\;.,{\hat{pf}}_{ͷm}\right)$$

$$=\left(\begin{array}{c} \left(1-\left(\prod_{ԟ=1}^{m}\prod_{ϛ=1}^{ͷ}{\left(1-\left(1-{\left(1-G_{ϛԟ}\right)}^{o_{ԟ}f_{ϛ}}\right)\right)}^{\frac{1}{mͷ}}\right)\right),\\ 1-\left(1-\prod_{ԟ=1}^{m}\prod_{ϛ=1}^{ͷ}{\left(1-\left(1-O_{ϛԟ}^{o_{ԟ}f_{ϛ}}\right)\right)}^{\frac{1}{mͷ}}\right),\\ 1-\left(1-\prod_{ԟ=1}^{m}\prod_{ϛ=1}^{ͷ}{\left(1-\left(1-{ȿ}_{ϛԟ}^{o_{ԟ}f_{ϛ}}\right)\right)}^{\frac{1}{mͷ}}\right) \end{array}\right)$$

$$=\left(\begin{array}{c} 1-\left(\prod_{ԟ=1}^{m}\prod_{ϛ=1}^{ͷ}{\left({\left(1-G_{ϛԟ}\right)}^{o_{ԟ}f_{ϛ}}\right)}^{\frac{1}{mͷ}}\right),\\ 1-\left(1-\prod_{ԟ=1}^{m}\prod_{ϛ=1}^{ͷ}{\left(O_{ϛԟ}^{o_{ԟ}f_{ϛ}}\right)}^{\frac{1}{mͷ}}\right),\\ 1-\left(1-\prod_{ԟ=1}^{m}\prod_{ϛ=1}^{ͷ}{\left({ȿ}_{ϛԟ}^{o_{ԟ}f_{ϛ}}\right)}^{\frac{1}{mͷ}}\right) \end{array}\right)$$

$$=\left(\frac{1}{mͷ}\begin{array}{c} \begin{array}{c} m\\ \;⊕ \end{array}\\ ԟ=1 \end{array}\begin{array}{c} \begin{array}{c} ͷ\\ \;⊕ \end{array}\\ ϛ=1 \end{array}\left(o_{ԟ}\left(f_{ϛ}\hat{pf_{ϛԟ}}\right)\right)\right)$$

Case 4 If r=s=1, then $WPFS_{ft}BM$ will degenerate into weighted $PFS_{ft}$ interrelated square mean

$$WPFS_{ft}BM^{1,1}\left({\hat{pf}}_{11},{\hat{pf}}_{12},.\;.\;.,{\hat{pf}}_{ͷm}\right)={\left(\frac{1}{mͷ\left(m-1\right)\left(ͷ-1\right)}\begin{array}{c} \begin{array}{c} m\\ \;⊕ \end{array}\\ \begin{array}{c} ԟ,ᶅ=1\\ ԟ\neq ᶅ \end{array} \end{array}\begin{array}{c} \begin{array}{c} ͷ\\ \;⊕ \end{array}\\ \begin{array}{c} ϛ,Ѯ=1\\ ϛ\neq Ѯ \end{array} \end{array}\left(o_{ԟ}\left(f_{ϛ}\hat{pf_{ϛԟ}}\right)\right)⨂\left(o_{ᶅ}\left(f_{Ѯ}\hat{pf_{Ѯᶅ}}\right)\right)\right)}^{\frac{1}{2}}$$

$$=\left(\begin{array}{c} {\left(1-\left(\prod_{\begin{array}{c} ԟ,ᶅ=1\\ ԟ\neq ᶅ \end{array}}^{m}\prod_{\begin{array}{c} ϛ,Ѯ=1\\ ϛ\neq Ѯ \end{array}}^{ͷ}{\left(1-\left(1-{\left(1-G_{ϛԟ}\right)}^{o_{ԟ}f_{ϛ}}\right)\left(1-{\left(1-G_{Ѯᶅ}\right)}^{o_{ᶅ}f_{Ѯ}}\right)\right)}^{\frac{1}{mͷ\left(m-1\right)\left(ͷ-1\right)}}\right)\right)}^{\frac{1}{2}},\\ 1-{\left(1-\prod_{\begin{array}{c} ԟ,ᶅ=1\\ ԟ\neq ᶅ \end{array}}^{m}\prod_{\begin{array}{c} ϛ,Ѯ=1\\ ϛ\neq Ѯ \end{array}}^{ͷ}{\left(1-\left(1-O_{ϛԟ}^{o_{ԟ}f_{ϛ}}\right)\left(1-O_{Ѯᶅ}^{o_{ᶅ}f_{Ѯ}}\right)\right)}^{\frac{1}{mͷ\left(m-1\right)\left(ͷ-1\right)}}\right)}^{\frac{1}{2}},\\ 1-{\left(1-\prod_{\begin{array}{c} ԟ,ᶅ=1\\ ԟ\neq ᶅ \end{array}}^{m}\prod_{\begin{array}{c} ϛ,Ѯ=1\\ ϛ\neq Ѯ \end{array}}^{ͷ}{\left(1-\left(1-{ȿ}_{ϛԟ}^{{oq}_{ԟ}f_{ϛ}}\right)\left(1-{ȿ}_{Ѯᶅ}^{o_{ᶅ}f_{Ѯ}}\right)\right)}^{\frac{1}{mͷ\left(m-1\right)\left(ͷ-1\right)}}\right)}^{\frac{1}{2}} \end{array}\right)$$

## 6 Algorithm

In this section, we will study the algorithm based on the introduced technique. Also, we will provide an illustrative example to support our work.

Assume that $ⱬ=\left\{{ⱬ}_{1},{ⱬ}_{2},.\;.\;.,{ⱬ}_{r}\right\}$ be the set of different alternatives and $H=\left\{h_{1},h_{2},.\;.\;.\;.,h_{m}\right\}$ be the set of parameters whose WVs is $o_{ԟ}\left(ԟ=1,2,.\;.\;.\;.,m\right)$ with constraint that $\sum_{ϛ=1}^{ͷ}o_{ԟ}=1$. Suppose $S=\left\{g_{1},g_{2},.\;.\;.\;.,g_{ͷ}\right\}$ denote a set of experts with WVs $f_{ϛ}=\left(ϛ=1,2,.\;.\;.,ͷ\right)$ with condition that $\sum_{ϛ=1}^{ͷ}f_{ϛ}=1$. Consider experts provide their assessment in the shape of $S_{ft}Ns$ ${\hat{pf}}_{ϛԟ}=\left\langle G_{ϛԟ},O_{ϛԟ},{ȿ}_{ϛԟ}\right\rangle$. Now the stepwise algorithm is given below

Step 1 Gather data for each alternative in the shape of $PFS_{ft}$ matrix $M={\hat{pf}}_{ϛԟ}={\left\langle G_{ϛԟ},O_{ϛԟ},{ȿ}_{ϛԟ}\right\rangle }_{ͷ\times m}$ as given below

$$Q=\left[\begin{array}{cccc} {\hat{pf}}_{11} & {\hat{pf}}_{12} & \cdots & {\hat{pf}}_{1m}\\ {\hat{pf}}_{21} & {\hat{pf}}_{22} & \cdots & {\hat{pf}}_{2m}\\ \vdots & \vdots & \ddots & \vdots \\ {\hat{pf}}_{ͷ1} & {\hat{pf}}_{ͷ2} & \cdots & {\hat{pf}}_{ͷm} \end{array}\right]$$

Step 2 Normalize the decision matrix by utilizing the formula given as

$$r_{ϛԟ}=\left\{ \begin{array}{c} {\hat{pf}}_{ϛԟ}\;;\;h_{ԟ}\in cost\;type\\ {\left({\hat{pf}}_{ϛԟ}\right)}^{c}\;;\;h_{ԟ}\in benefit\;type \end{array}\right.$$

Where ${\left({\hat{pf}}_{ϛԟ}\right)}^{c}$ is the complement of ${\hat{pf}}_{ϛԟ}=\left\langle G_{ϛԟ},O_{ϛԟ},{ȿ}_{ϛԟ}\right\rangle$.

Step 3 Use $PFS_{ft}BM$ or $WPFS_{ft}BM$ operators to aggregate $PFS_{ft}Ns\;r_{ϛԟ}\left(ϛ=1,2,.\;.\;.ͷ;ԟ=1,2,.\;.\;.,m\right)$ for each alternative ${ⱬ}_{b}\left(b=1,2,.\;.\;.\;.,r\right)$ into the collective decision matrix $Q_{b}\text{.}$.
Step 4 Find out the score value for each alternative by using equation (2). Note that if score values are the same then we will use equation (3) to deduce the result.
Step 5 Rank the alternatives ${ⱬ}_{b}\left(b=1,2,.\;.\;.\;.,r\right)$ and select the best result.

### 6.1 Application in medical diagnosis

Here in this part, we will elaborate on the numerical analysis of initiated work to support the given approach.

Example 3 Consider a team of four doctors $D_{1},D_{2},D_{3}\;and\;D_{4}$ having WVs {0.20,0.30,0.32,0.18}, will give their evaluation of the rapid increase of four different cancer diseases in the world like $C_{1}=Prostate,C_{2}=Lungs\;cancer,C_{3}=Colon\;and\;rectum,C_{4}=Breast\;cancer$. Suppose the corresponding set of parameters is given by $\left\{h_{1}=High\;body\;mass\;index,h_{2}=Use\;of\;tobacco,h_{3}=Low\;fruit\;and\;vegetable\;intake\right\}\text{.}$ Also, assume that WVs for parameters are {0.24,0.34,0.42}. Assume that experts come up with their assessments in the term of $PFS_{ft}Ns\text{.}$ Now, the stepwise procedure is given below

Step 1 The specialist supplies their evaluation data in the shape of $PFS_{ft}Ns$ as given in Table 2, Table 3, Table 4, Table 5

Step 2 Assume that all the data is of the same kind, so there is no need for normalizing the information given in Table 2, Table 3, Table 4, Table 5

Step 3 Now we use $WPFS_{ft}BM$ aggregation operator to aggregate the preference of these values corresponding to each alternative. For simplicity assume that r=s=1. We get

$$r_{1}=\left(0.6607,0.0588,0.0605\right),r_{2}=\left(0.6626,0.0430,0.0683\right)$$

$$r_{3}=\left(0.6722,0.0312,0.0380\right),r_{4}=\left(0.6778,0.0571,0.00387\right)$$

Step 4 Now we use the definition of the score function to calculate the score values

$$Sco.\left(r_{1}\right)=0.6002,Sco.\left(r_{2}\right)=0.5943$$

$$Sco.\left(r_{3}\right)=0.6342,Sco.\left(r_{4}\right)=0.6391$$

Step 5 Now according to the results of Step 4 we note that $C_{4}=Breast\;cancer$ is a more rapidly increasing disease in the world.

**Table 2:** Picture fuzzy soft information for $C_{1}$.

	$h_{1}$	$h_{2}$	$h_{3}$
$D_{1}$	(0.21,0.34,0.33)	(0.10,0.41,0.31)	(0.11,0.29,0.27)
$D_{2}$	(0.51,0.11,0.22)	(0.21,0.20,0.51)	(0.21,0.22,0.23)
$D_{3}$	(0.13,0.23,0.41)	(0.16,0.25,0.12)	(0.34,0.41,0.19)
$D_{4}$	(0.42,0.11,0.26)	(0.54,0.14,0.18)	(0.5,0.12,0.11)

**Table 3:** Picture fuzzy soft information $C_{2}$.

	$h_{1}$	$h_{2}$	$h_{3}$
$D_{1}$	(0.25,0.30,0.40)	(0.45,0.17,0.23)	(0.12,0.25,0.15)
$D_{2}$	(0.27,0.25,0.35)	(0.10,0.20,0.45)	(0.11,0.10,0.42)
$D_{3}$	(0.42,0.11,0.10)	(0.38,0.32,0.26)	(0.40,0.25,0.30)
$D_{4}$	(0.35,0.23,0.30)	(0.25,0.15,0.12)	(0.12,0.16,0.26)

**Table 4:** Picture fuzzy soft information $C_{3}$.

	$h_{1}$	$h_{2}$	$h_{3}$
$D_{1}$	(0.19,0.26,0.34)	(0.12,0.09,0.26)	(0.25,0.24,0.16)
$D_{2}$	(0.34,0.13,0.23)	(0.22,0.17,0.16)	(0.12,0.10,0.10)
$D_{3}$	(0.28,0.29,0.30)	(0.12,0.15,0.13)	(0.18,0.19,0.20)
$D_{4}$	(0.42,0.11,0.19)	(0.14,0.16,0.17)	(0.21,0.22,0.23)

**Table 5:** Picture fuzzy soft information $C_{4}$.

	$h_{1}$	$h_{2}$	$h_{3}$
$D_{1}$	(0.22,0.10,0.24)	(0.12,0.14,0.13)	(0.18,0.20,0.21)
$D_{2}$	(0.12,0.21,0.32)	(0.15,0.17,0.16)	(0.12,0.19,0.18)
$D_{3}$	(0.20,0.13,0.12)	(0.19,0.18,0.20)	(0.15,0.21,0.25)
$D_{4}$	(0.23,0.23,0.19)	(0.21,0.13,0.23)	(0.33,0.28,0.12)

## 7 Comparative analysis

Here in this part, we have to investigate the comparative study of initiated operators with some prevailing theories to support our work.

**Example 4:** Suppose we have three alternatives $al_{1},al_{2},and\;al_{3}$ which are to be evaluated by four experts $\tau_{1},\tau_{2},\tau_{3}\;and\;\tau_{4}$ with WVs {0.19,0.31,0.17,0.33} corresponding to a set of the parameter $\left\{\varepsilon_{1},\varepsilon_{2},\varepsilon_{3}\right\}$ having WVs {0.26,0.35,0.39}. Suppose experts provide their assessment of each alternative in the shape of $PFS_{ft}Ns$ given in Table 6, Table 7, Table 8. We compare our work with Xu and Yager method [14], Garg and Arora method [37], the Liang et al. method [19], Liu and Liu method [28], and Ates and Akay method [42]. The overall results are given in Table 9.

**Table 6:** Picture fuzzy soft information for alternative $al_{1}$.

	$\varepsilon_{1}$	$\varepsilon_{2}$	$\varepsilon_{3}$
$\tau_{1}$	(0.12,0.33,0.24)	(0.32,0.44,0.15)	(0.19,0.34,0.27)
$\tau_{2}$	(0.51,0.11,0.22)	(0.15,0.28,0.38)	(0.17,0.44,0.15)
$\tau_{3}$	(0.31,0.43,0.21)	(0.38,0.28,0.33)	(0.29,0.27,0.13)
$\tau_{4}$	(0.14,0.21,0.53)	(0.45,0.21,0.13)	(0.12,0.35,0.45)

**Table 7:** Picture fuzzy soft information for alternative $al_{2}$.

	$\varepsilon_{1}$	$\varepsilon_{2}$	$\varepsilon_{3}$
$\tau_{1}$	(0.22,0.13,0.14)	(0.33,0.34,0.26)	(0.19,0.23,0.22)
$\tau_{2}$	(0.41,0.12,0.32)	(0.13,0.12,0.23)	(0.15,0.41,0.25)
$\tau_{3}$	(0.51,0.11,0.19)	(0.28,0.14,0.43)	(0.21,0.32,0.16)
$\tau_{4}$	(0.24,0.29,0.18)	(0.35,0.26,0.13)	(0.14,0.25,0.35)

**Table 8:** Picture fuzzy soft information for alternative $al_{3}$.

	$\varepsilon_{1}$	$\varepsilon_{2}$	$\varepsilon_{3}$
$\tau_{1}$	(0.21,0.35,0.27)	(0.13,0.14,0.25)	(0.21,0.28,0.17)
$\tau_{2}$	(0.15,0.14,0.37)	(0.25,0.12,0.23)	(0.37,0.24,0.19)
$\tau_{3}$	(0.2,0.3,0.4)	(0.37,0.18,0.2)	(0.22,0.32,0.3)
$\tau_{4}$	(0.24,0.25,0.33)	(0.54,0.11,0.1)	(0.2,0.3,0.15)

**Table 9:** Overall evaluation results.

Methods	Score Values	Ranking results
Xu and Yager Method [14]	Cannot handle given data	No result
Garg and Arora Method [37]	Cannot handle given data	No result
Liang et al. Method [19]	Cannot handle given data	No result
Liu and Liu Method [28]	Cannot handle given data	No result
Ates and Akay Method [42]	$Sco.\left(al_{1}\right)=0.3214\text{,}$ $Sco.\left(al_{2}\right)=0.5124\text{,}$ $Sco.\left(al_{3}\right)=0.5432\text{,}$	$al_{3}>al_{2}>al_{1}$
$PFS_{ft}BM$ operators (Proposed)	$Sco.\left(al_{1}\right)=-0.5185\text{,}$ $Sco.\left(al_{2}\right)=-0.4182\text{,}$ $Sco.\left(al_{3}\right)=-0.4073\text{,}$	$al_{3}>al_{2}>al_{1}$
$WPFS_{ft}BM$ operators (Proposed)	$Sco.\left(al_{1}\right)=0.5129\text{,}$ $Sco.\left(al_{2}\right)=0.5538\text{,}$ $Sco.\left(al_{3}\right)=0.5620\text{,}$	$al_{3}>al_{2}>al_{1}$

From the analysis of the above-given results, we can see that.

1. The method proposed by Xu and Yager [14], Garg and Arora [37], Liang et al. [19] and Liu and Liu method [28] can either deal with intuitionistic fuzzy, Pythagorean fuzzy, or q-rung orthopair fuzzy information, while the data given in Table 6, Table 7, Table 8 consist of picture fuzzy soft information. So, all the above-mentioned methods cannot handle such type of information because all above methods lack abstinence grade in their structure which is a weak property for these methods. In another way proposed picture fuzzy soft Bonferroni means operators can deal with such type of information because picture fuzzy structure can consider the abstinence grade and this property make our initiated work more superior to other existing notions.
2. Also, note that although Ates and Akay method [42] can consider picture fuzzy data and this structure lacks the property of parameterization. Because the parameterization tool is one of the most familiar tools in soft set theory that makes the soft set more general than that of the fuzzy structures. That is the reason that our work is more efficient because it provides the facility to all those decision-makers who want to consider parameterization toll in their decisions.
3. Note that the best alternative in all cases is the same which shows the reliability and authenticity of the introduced picture fuzzy Bonferroni mean operators.

## 8 Conclusion

Picture fuzzy soft set is a dominant structure in fuzzy set theory that can consider the three types of aspects like yes, no, and abstain in one structure. Moreover, picture fuzzy soft sets can consider the parameterization tool that can help and generalize many fuzzy set theories. So, based on the dominant features of the picture fuzzy soft set, in this article, we have initiated the study of $PFS_{ft}BM$ aggregation operators and $WPFS_{ft}BM$ aggregation operators. Moreover, characteristic analysis of the proposed aggregation operators has been investigated. Furthermore, a decision-making algorithm along with an explanatory example is introduced to assist our work. A comparative study is also given that can help to examine how these introduced notions are bounded and how this introduced work is more general.

We can see that existing notions are limited because the developed notion uses the condition that sum(MG,AG,NMG)∈[0,1]. But if the decision maker comes up with spherical fuzzy soft data like 0.5 as MG, 0.4 as AG and 0.6 as NMG then the basic condition for developed theory is violated because we can see that sum(0.5,0.4,0.6)∉[0,1]. So existing notions fail to handle spherical fuzzy soft data. Similarly when decision-makers come up with T-spherical fuzzy soft information so we can observe that T-spherical fuzzy soft data use the condition that $sum\left({MG}^{q},{AG}^{q},{NMG}^{q}\right)\in \left[0,1\right]$ for q≥1. So T-spherical fuzzy soft data cannot be covered by the existing notions.

Moreover, this idea can be extended to a spherical fuzzy soft rough set as proposed in Ref. [45]. We can introduce some hybrid notions as proposed in Ref. [46]. We can also extend these notions to complex picture fuzzy soft set theory [47]. Moreover, these notions can be extended to complex fuzzy soft set theory [48] and bipolar complex intuitionistic fuzzy soft set theory [49].

## Authorship contribution

XiaopenPg Yang: conceived and designed the experiments; wrote the paper.

Tahir Mahmood: performed the experiments; contributed reagents, materials, analysis tools or data.

Jabbar Ahmmad: performed the experiments; analyzed and interpreted the data; wrote the paper.

## Data availability

No data was used for the research described in the article.

## Declaration of competing interest

The authors declare that they have no known competing financial interests or personal relationships that could have appeared to influence the work reported in this paper.
